# The causal relationship between serum metabolites and the risk of psoriasis: a Mendelian randomization and meta-analysis study

**DOI:** 10.3389/fimmu.2024.1343301

**Published:** 2024-03-11

**Authors:** Yujie Yang, Xuwei Zheng, Haiying Lv, Bin Tang, Yiyuan Zhong, Qianqian Luo, Yang Bi, Kexin Yang, Haixin Zhong, Haiming Chen, Chuanjian Lu

**Affiliations:** ^1^The Second Clinical College of Guangzhou University of Chinese Medicine, Guangzhou, China; ^2^State Key Laboratory of Dampness Syndrome of Chinese Medicine, The Second Affiliated Hospital of Guangzhou University of Chinese Medicine (Guangdong Provincial Hospital of Chinese Medicine), Guangzhou, China; ^3^Guangdong Provincial Key Laboratory of Clinical Research on Traditional Chinese Medicine Syndrome, Guangzhou, China; ^4^Guangdong Provincial Clinical Medicine Research Center for Chinese Medicine Dermatology, Guangzhou, China; ^5^Guangdong-Hong Kong-Macau Joint Lab on Chinese Medicine and Immune Disease Research, Guangzhou University of Chinese Medicine, Guangzhou, China

**Keywords:** psoriasis, Mendelian randomization, metabolites, causal effect, implication

## Abstract

**Objective:**

To explore the influence of serum metabolites on the risk of psoriasis.

**Methods:**

In the initial stage, we applied Mendelian randomization to evaluate the association between 1,400 serum metabolites and the risk of psoriasis. Causal effects were primarily assessed through the Inverse-Variance Weighted method and Wald Ratio’s odds ratios, and 95% confidence intervals. False Discovery Rate was used for multiple comparison corrections. Sensitivity analyses were conducted using Cochran’s Q Test, MR-PRESSO. MR-Steiger Test was employed to check for reverse causality. In the validation stage, we sought other sources of psoriasis GWAS data to verify the initial results and used meta-analysis to combine the effect sizes to obtain robust causal relationships. In addition, we also conducted metabolic pathway enrichment analysis on known metabolites that have a causal relationship with the risk of psoriasis in both stages.

**Results:**

In the initial stage, we identified 112 metabolites causally associated with psoriasis, including 32 metabolite ratios and 80 metabolites (69 known and 11 unknown). In the validation stage, 24 metabolites (16 known, 1 unknown, and 7 metabolite ratios) were confirmed to have a causal relationship with psoriasis onset. Meta-analysis results showed that the overall effect of combined metabolites was consistent with the main analysis in direction and robust in the causal relationship with psoriasis onset. Of the 16 known metabolites, most were attributed to lipid metabolism, with 5 as risk factors and 8 as protective factors for psoriasis. Peptidic metabolite Gamma-glutamylvaline levels had a negative causal relationship with psoriasis, while exogenous metabolite Catechol sulfate levels and amino acid 3-methylglutaconate levels had a positive causal relationship with the disease onset. The metabolites associated with psoriasis risk in the two stages are mainly enriched in the following metabolic pathways: Glutathione metabolism, Alpha Linolenic Acid and Linoleic Acid Metabolism, Biosynthesis of unsaturated fatty acids, Arachidonic acid metabolism, Glycerophospholipid metabolism.

**Conclusion:**

Circulating metabolites may have a potential causal relationship with psoriasis risk, and targeting specific metabolites may benefit psoriasis diagnosis, disease assessment, and treatment.

## 1 Introduction

Psoriasis is a chronic inflammatory disease that affects the skin and joints and is now considered a systemic condition due to its frequent association with multiple systemic disorders. Metabolic Syndrome (MetS) is the most common comorbidity of psoriasis and a risk factor for cardiovascular disease, representing a principal cause of mortality in patients with psoriasis (1). The prevalence of MetS in patients with psoriasis ranges from 20% to 50%, and it increases with the severity of the psoriasis condition (2, 3).

Multiple studies employing metabolomics have identified a metabolic profile in the plasma of psoriasis patients, uncovering extensive metabolic disturbances in lipids and amino acids among these individuals (4–6). Chen et al. observed significant alterations in the metabolism of amino acids and carnitines in patients with psoriasis, particularly involving the metabolism of amino acids, branched-chain amino acids, and carbon monoxide (5). Zeng et al. found significant differences in the components of glycerophospholipid metabolism, such as lysophosphatidic acid (LPA), lysophosphatidylcholine (LysoPC), phosphatidic acid (PA), phosphatidylinositol (PI), and phosphatidylcholine (PC), between the plasma of psoriasis patients and healthy individuals (4). The concurrence of psoriasis and metabolic dysregulation is increasingly becoming a significant public health issue, yet the pathomechanisms of their comorbidity remain unclear.

Although a definitive causal relationship has not been established, genetic susceptibility, common inflammatory pathways, and environmental factors may contribute to the metabolic abnormalities observed in patients with psoriasis (1). In the field of genetics research, numerous single nucleotide polymorphisms have been identified in proximity to loci associated with innate and adaptive immune genes relevant to psoriasis, such as antigen-presenting genes (HLA-C, ERAP1) and Th17 cell activation genes (IL23R, IL23A, IL12B, TRAF3IP2) (7). Alterations in metabolic processes can lead to epigenetic abnormalities. Therefore, studying differential metabolic biomarkers in psoriasis is of significance for identifying potential therapeutic targets for the disease.

Mendelian randomization (MR) is an epidemiological methodology that utilizes genetic variants, primarily single nucleotide polymorphisms (SNPs), as instrumental variables (IVs) to proxy for target exposure variables, with the aim of assessing the causal relationship between exposures and specific outcomes (8). Published Genome-Wide Association Studies have integrated the associations between circulating metabolites and SNPs, facilitating the causal inference of the relationship between metabolic products and the risk of psoriasis. Consequently, this study employs Mendelian Randomization to explore the causal link between circulating metabolites and psoriasis risk from a genetic variation perspective, aiming to identify potential metabolites related to the onset of psoriasis in hopes of discovering targets for disease activity assessment, diagnosis, and treatment.

## 2 Subjects and methods

### 2.1 Study design

**Figure 1:**
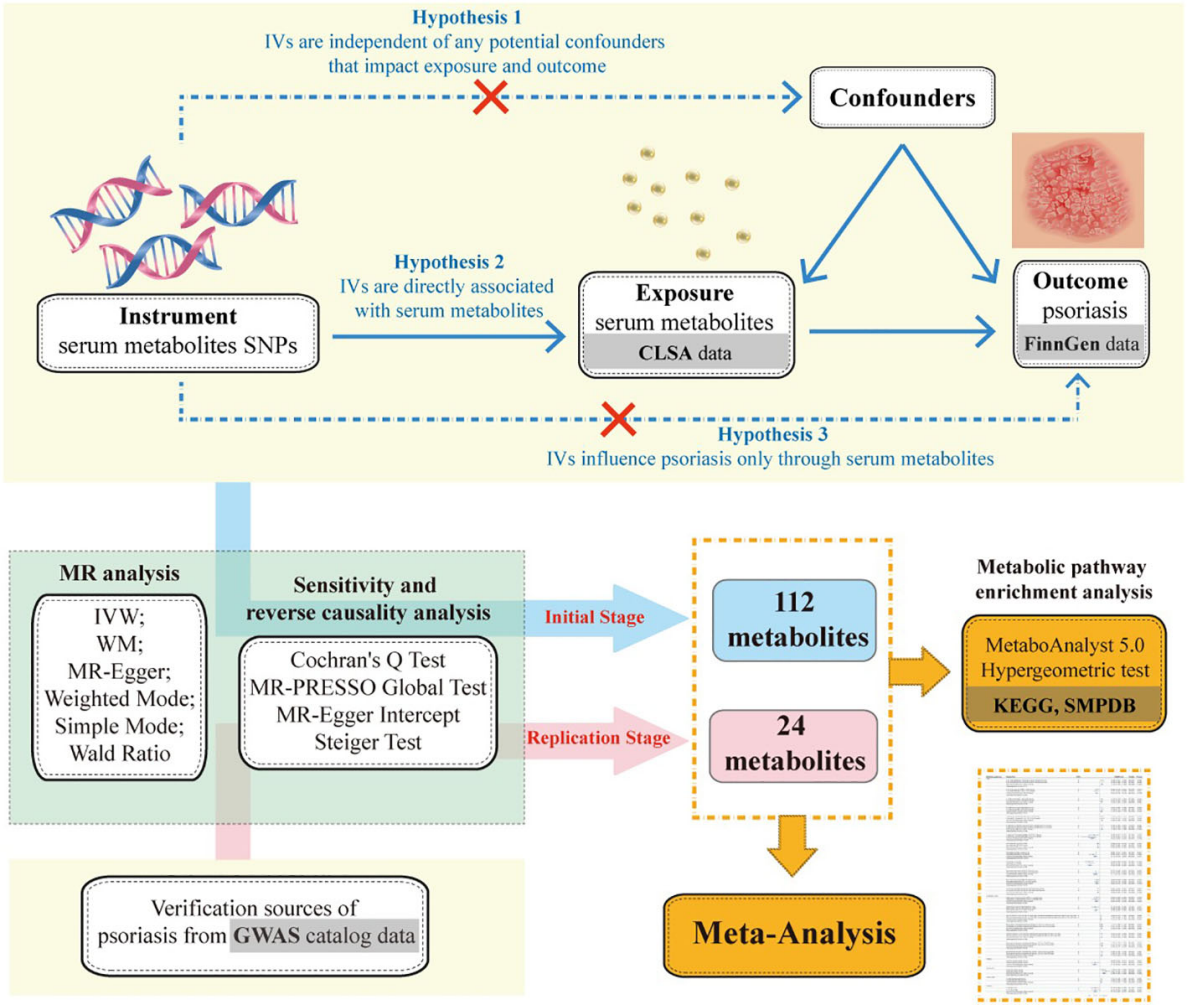
Mendelian randomization’s three hypotheses and research flowchart.

To elucidate the causal relationship between serum metabolites and the risk of developing psoriasis, we embarked on this MR study. Initially, we employed a two-sample MR approach to screen for causal associations between 1,400 metabolites and psoriasis. We also conducted multiple sensitivity analyses to ensure the robustness of our findings. Subsequently, we validated the results from the initial stage using psoriasis GWAS data from other sources. Lastly, we applied a meta-analysis to integrate effect sizes of metabolites with causal effects on both psoriasis outcomes, thereby obtaining a robust causal inference (**Figure 1**).

### 2.2 Data source

The summary statistics for serum metabolites utilized in this study were sourced from The Canadian Longitudinal Study of Aging (CLSA) (9). The study conducted genome-wide genotyping and circulating plasma metabolite assessments on 8,299 unrelated European individuals. Following stringent pre-GWAS genotype quality control, approximately 15.4 million SNPs were retained for GWAS assessment. The levels of 1,458 metabolites in plasma samples were quantified using the Ultrahigh Performance Liquid Chromatography-Tandem Mass Spectroscopy (UPLC-MS/MS) platform. After standardization, which involved the removal of entities representing systemic artifacts, misassignments, and background noise, a rigorous quality control process ascertained 1,091 metabolites (850 known substances and 241 unknown entities) and 309 metabolite ratios for inclusion in the genome-wide association analyses. The 850 metabolites with established identities were categorized into eight super-pathways: carbohydrates, amino acids, nucleotides, vitamins, lipids, peptides, energy metabolism products, and xenobiotics. Given that many metabolites serve as substrates and products of enzymatic reactions, investigating the genetic determinants of substrate-product ratios can enhance the understanding of broader biological processes; hence, the 309 metabolite ratios were also incorporated into this study.

The initial stage outcome data were derived from the FinnGen biobank analysis consortium, comprising 9,267 psoriasis patients and 364,071 healthy controls (10). Validation data were sourced from a study published by Stuart PE in 2021, including 15,976 psoriasis cases and 28,194 healthy controls (11). The genetic data utilized in this study conform to ethical standards and have been approved by local ethics review committees, representing legally public research data.

### 2.3 Selection of instrumental variables

Instrumental variables were selected through the following steps. (1) Genetic variants associated with metabolic traits were identified at a genome-wide significance threshold set at *P*< 5×10^-7^. (2) The independence of the aggregated SNPs was assessed based on linkage disequilibrium, selecting instrument variables without linkage effects in the metabolic data (parameters set at r^2^< 0.01 within a 5000 kb distance). (3) These instrumental variables were then extracted from the psoriasis dataset, excluding any IVs related to the outcomes and discarding palindromic SNPs. (4) The strength of individual SNPs was tested by calculating the F-statistic, retaining those with an *F*-statistic > 10.The *F*-statistic is calculated using the formula $F=\frac{R^{2}\times \left(N-K-1\right)}{K\left(1-R^{2}\right)}$ , where R^2^ is the proportion of variance in the exposure that is explained by the instrumental variables, given by ${\text{R}}^{2}=\frac{2\times EAF\times \left(1-EAF\right)\times \beta^{2}}{2\times EAF\times \left(1-EAF\right)\times \beta^{2}+2\times EAF\times \left(1-EAF\right)\times N\times SE^{2}}$ . Within this formula, *N* denotes the sample size for the exposure, *K* represents the number of instrumental variables, *EAF* is the effect allele frequency, β is the effect size, and *SE* is the standard error of the effect size (12, 13).

### 2.3 Two-sample Mendelian randomization

The present study primarily employs the inverse-variance weighted (IVW) method, weighted median (WM), MR-Egger, Weighted mode, simple mode, and Wald Ratio for analysis. The IVW method, assuming all SNPs are valid and independent, constrains the regression intercept to zero and uses the reciprocal of the outcome variance as weights for fitting (14). When each genetic variant satisfies the assumptions of an instrumental variable, IVW is presumed to provide the most accurate results; hence, the IVW outcomes are often regarded as the major standard for the assessment of causal effects (14, 15). In this study, the *P*-value of IVW is used as the primary indicator for assessing the causal effect between exposure and outcome. If only a single instrumental variable is obtained, the *P*-value from the Wald Ratio is used for assessment. Other methods serve to supplement the evaluation of MR results, and a consistent direction of effect size (*β*-value) across different methods indicates robust findings. The False Discovery Rate (FDR) correction is applied to adjust for multiple testing of all *P*-value obtained from IVW and Wald Ratio methods.*P*valueof FDR less than 0.05 indicates a clear causal relationship between exposure and outcome, while greater than 0.05 indicates a potential causal relationship.

### 2.4 Sensitivity analysis

Diverse methodologies are employed to evaluate the heterogeneity and pleiotropy of the results. Heterogeneity was tested by Cochran’s Q statistic where a *P*-value greater than 0.05 suggests an absence of heterogeneity (16). Pleiotropy can be evaluated using the MR-PRESSO test and the MR-Egger regression intercept; if the *P*-value for the MR-PRESSO global Test and the MR-Egger intercept are greater than 0.05, this suggests that pleiotropy is not present (17, 18). It is worth noting that when the number of SNPs is insufficient (3 for MR-PRESSO, 2 for MR-Egger), pleiotropy test will not be performed.

The Steiger test is used to examine the directionality of the instrumental variable’s effect on the outcome, avoiding reverse causation, thereby confirming whether the results support the initial hypothesis. *P*-value of Steiger greater than 0.05 for the instrumental variable indicates the presence of reverse causation (19, 20).

### 2.5 Replication stage and meta-analysis

We sought additional sources of outcome data for psoriasis and conducted MR validation on the initial stage results under the same conditions, retaining metabolites and metabolite ratios that still exhibited a causal effect with psoriasis. Subsequently, we merged the two MR results (odds ratios, OR; 95% confidence intervals, CI) through a meta-analysis. If the merged results showed heterogeneity, indicated by an I^2^ value greater than 50%, a random-effects model was utilized; otherwise, a fixed-effects model was applied. The statistical threshold for the meta-analysis was set at 0.05. The aforementioned study was primarily conducted using the Two Sample MR and meta packages within the R statistical software, version 4.2.1, with a significance level (alpha) of 0.05.

### 2.6 Metabolic pathway enrichment analysis

The HMDB IDs of known metabolites were retrieved from The Human Metabolome Database (https://hmdb.ca/), and enrichment analysis of the metabolic pathways associated with these metabolites was conducted using MetaboAnalyst 5.0 (https://www.metaboanalyst.ca/). The pathway libraries selected for this analysis were the Small Molecule Pathway Database (SMPDB) and the Kyoto Encyclopedia of Genes and Genomes (KEGG). The enrichment method employed was the Hypergeometric Test, and the significance level for metabolic pathway analysis was set at 0.01.

## 3 Results

### 3.1 Initial stage Mendelian randomization analysis results

**Figure 2:**
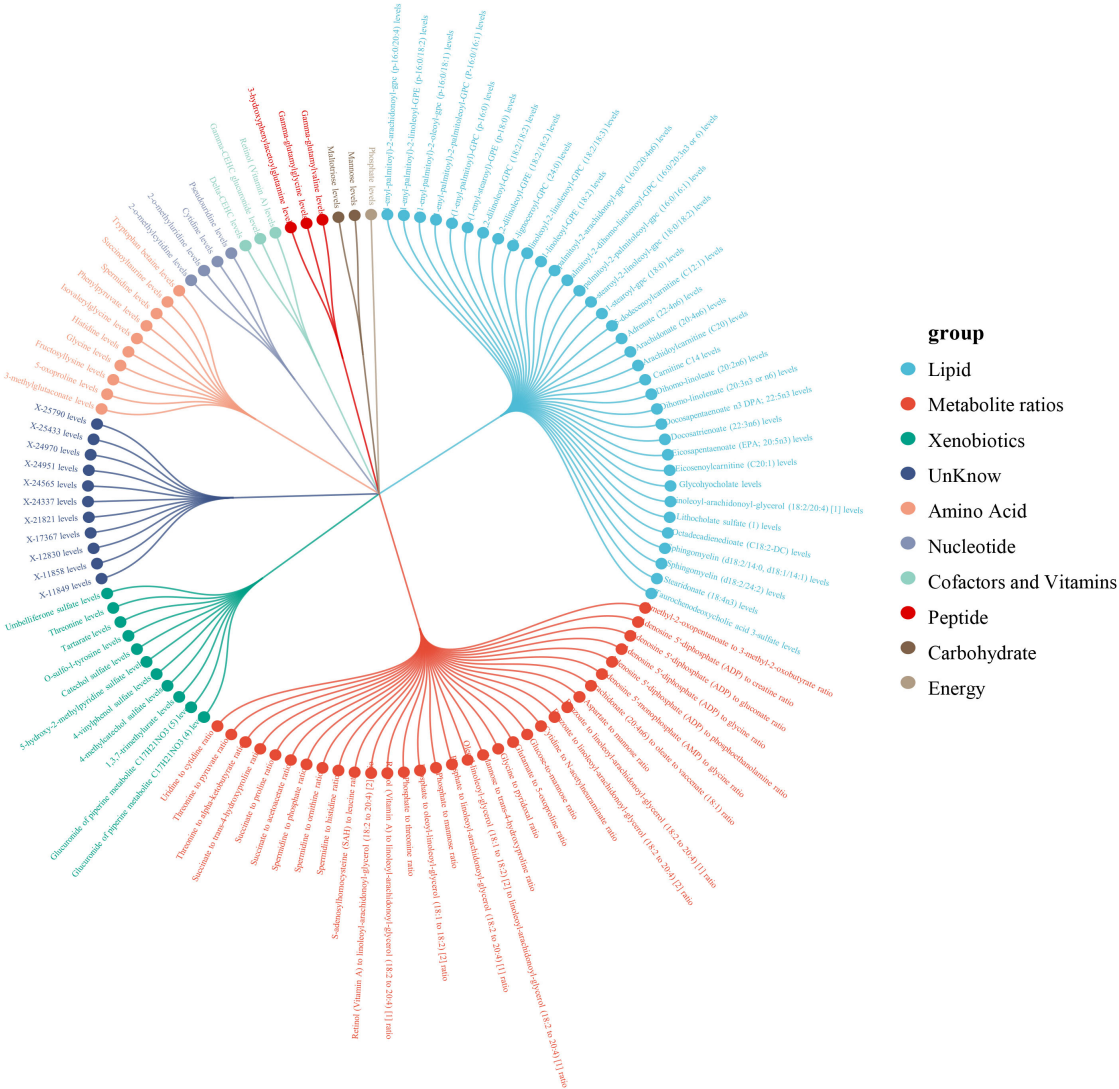
Classification of 112 metabolites.

Within a pool of 1,400 metabolites, 112 metabolites were discerned to have a causal association with psoriasis, including 32 metabolite ratios and 80 individual metabolites (of which 69 were identified metabolites and 11 were unidentified) (**Figure 2**). After adjustment for FDR, 7 metabolites or metabolite ratios remained significantly causally associated with psoriasis. Additionally, the 375 SNPs finally associated with psoriasis causality were all linked to strong instrumental variables (with an F-statistic > 10).Detailed information of IVs can be found in the **Supplementary Table 1**.

The 69 identified metabolites predominantly belonged to the categories of lipids, xenobiotics and amino acids. Of the 35 lipid metabolites analyzed, the strongest protective effect was identified as Carnitine C14 levels (OR: 0.49, 95% CI: 0.38-0.64, *P*_Wald Ratio_=2.43×10^-7^, *P*_FDR_=3.13×10^-4^), while the metabolite with the most potent risk effect was Dihomo-linoleate (20:2n6) levels (OR: 2.07, 95% CI: 1.00-4.28, *P*_Wald Ratio_ =0.049, *P*_FDR_=0.559).

Among the 11xenobiotic, the most significant positive and negative causal association with the onset of psoriasis was observed with elevated Umbelliferone sulfate levels (OR: 2.06, 95% CI: 1.53-2.79,*P*_Wald Ratio_=2.49×10^-6^, *P*_FDR_=0.002) and elevated 4-vinylphenol sulfate levels (OR: 0.53, 95% CI: 0.28-0.97, *P*_Wald Ratio_ = 0.041, *P*_FDR_= 0.538).

Among the 10amino acids metabolites, the increase in Spermidine levels exhibited the most significant positive causal effect on the incidence of psoriasis (OR: 1.37, 95%CI: 1.10-1.71, *P*_IVW_ = 0.005, *P*_FDR_=0.239), whereas the increase in Histidine levels was associated with the most significant negative causal effect (OR: 0.8, 95% CI: 0.69-0.92, *P*_IVW_=0.002, *P*_FDR_=0.192). Additionally, of the 32 metabolite ratios with a causal link to psoriasis, detailed information can be found in **Supplementary Table 2**.

**Figure 3:**
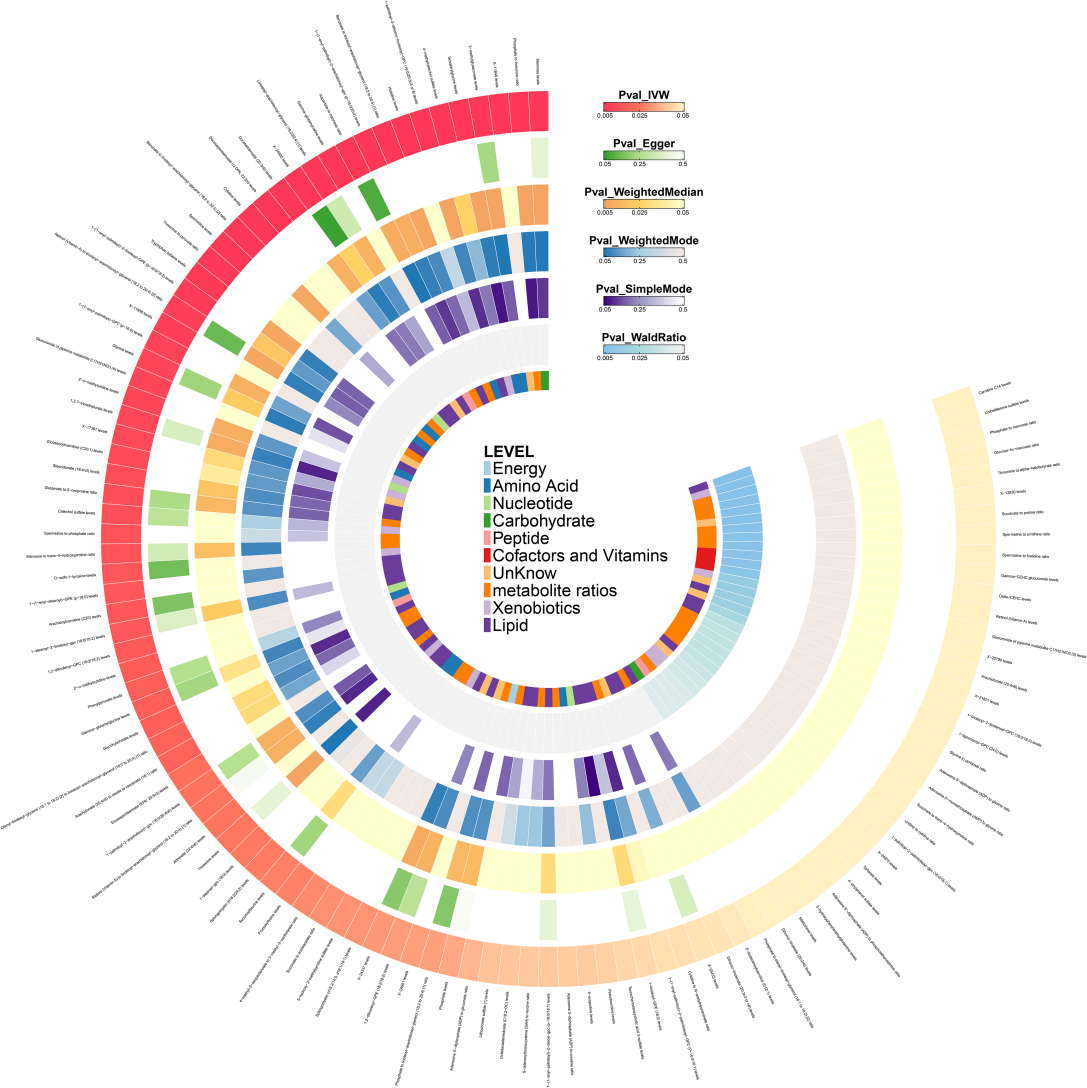
Heat map of the results of six Mendelian randomization analysis methods in the initial stage.

The direction of effect estimates for most metabolites was consistent across multiple analytical methods, rendering the results more reliable. Specific MR analysis results for different methods are presented in **Figure 3** and **Supplementary Table 2**.

### 3.2 Sensitivity and reverse causality analysis

3 metabolites were identified with heterogeneous results usedCochran’s Q test. Pleiotropy evaluation through the MR-PRESSO method detected horizontal pleiotropy in the exogenous metabolite Threonine levels. Detailed information is shown in **Supplementary Table 2**.

The MR-Steiger directionality test confirmed the accuracy of the exposure to outcome direction with all instrumental variables showing *P*_Steiger_< 0.05. P-values for testing the reverse relationship between each metabolite and psoriasis were all less than 0.05, suggesting no evidence of potential reverse causation. Detailed information is shown in **Supplementary Tables 2**, **3**.

### 3.3 Replication stage Mendelian randomization results and meta-analysis

**Figure 4:**
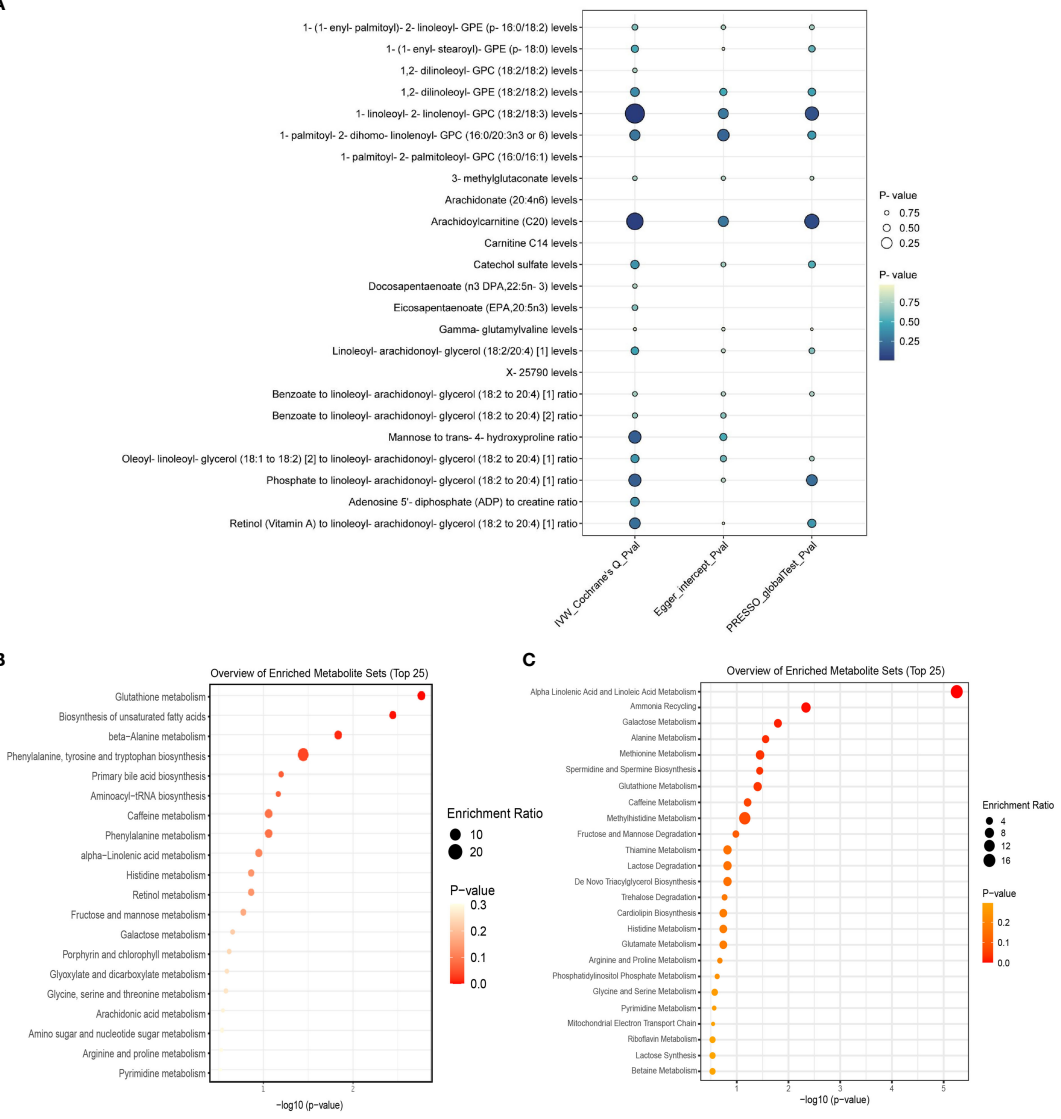
**(A)** displays the heterogeneity and pleiotropy analysis results of 24 metabolites that have been successfully validated. **(B)** displays the enrichment pathways of metabolites in KEGG and **(C)** displays the enrichment pathways of metabolites in SMPDE database.

**Figure 5:**
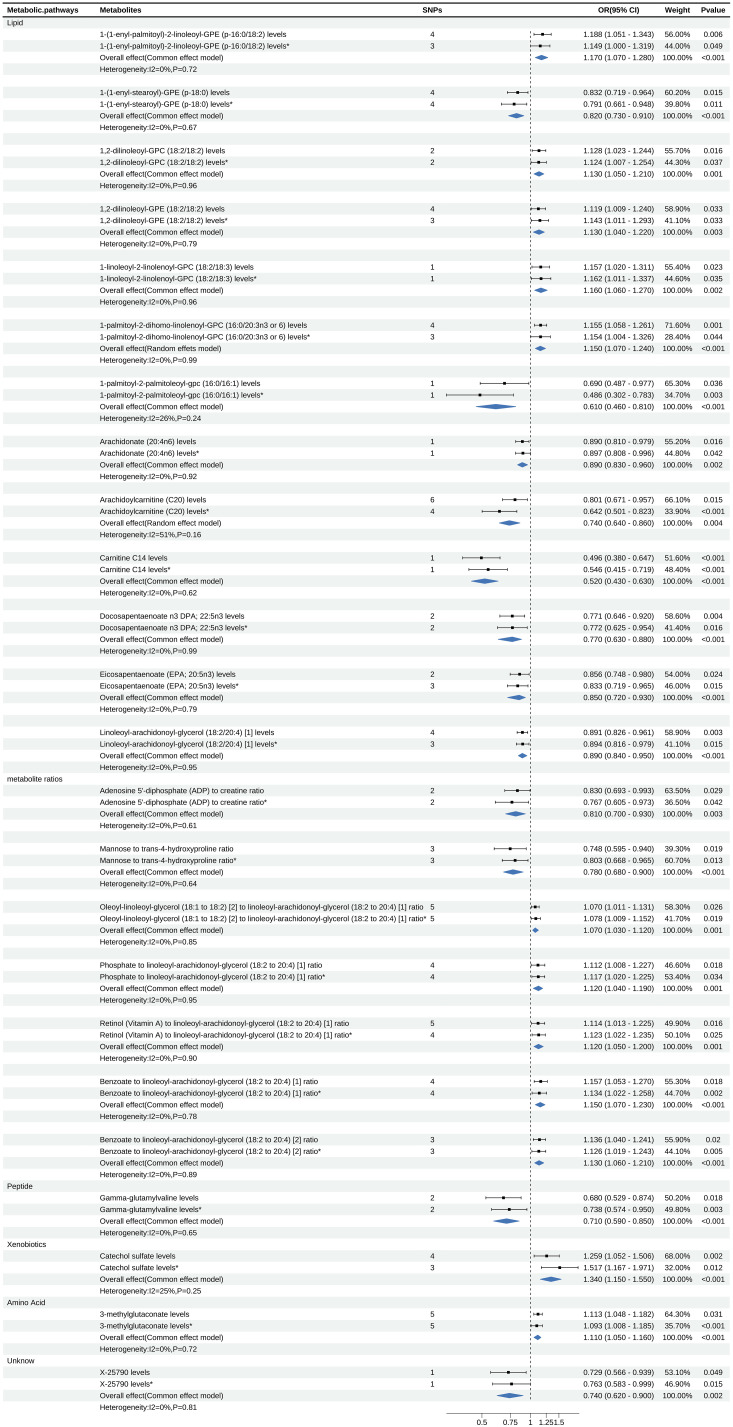
Forest map displays the results of Meta Analysis, where * is the result of Verification data.

Out of 112 metabolites with a putative causal relationship with psoriasis, 24 (7 metabolite ratios, 16 known metabolites, and 1 unknown metabolite) were successfully validated. **Figure 4A** visualizes the sensitivity analysis results of 24 metabolites. Detailed information of main MR Results of 24 metabolites are shown in the **Supplementary Table 4** and **Figure 5**.

For meta-analysis, heterogeneity test revealed heterogeneity in the metabolite Arachidoylcarnitine (C20) levels (I^2^ = 51%), while the rest of the metabolites showed no evidence of heterogeneity. The meta-analysis results indicated that the combined overall effect of the 24 metabolites was consistent with the direction of effect observed in two stages MR analysis and confirmed astable causal relationship between these metabolites and psoriasis (*P*<0.05).

Meta-analysis showed that among the 13 lipid metabolites, the increase of 1-(1-enyl-palmitoyl)-2-linoleoyl-GPE (p-16:0/18:2) was a strong risk factor for psoriasis (OR: 1.17, 95% CI: 1.07-1.28, *P*<0.001). Considering the insufficient phenotypic interpretation of a single SNP, the increase of Arachidoylcarnitine (C20) was a strong protective factor for psoriasis (OR: 0.74, 95% CI: 0.64-0.86, *P*=0.004). Additionally, the peptide metabolite Gamma-glutamylvaline levels were inversely associated with the risk of psoriasis (OR: 0.71, 95% CI: 0.59-0.85, *P*<0.001), while the xenobiotics Catechol sulfate levels (OR: 1.34, 95% CI: 1.15-1.55, *P*<0.001) and the amino acid metabolite 3-methylglutaconate levels (OR: 1.11, 95% CI: 1.05-1.16, *P*<0.001) were associated with an increased risk of psoriasis. Further details can be found in **Figure 5**.

### 3.4 Results of metabolic pathway enrichment analysis

We queried the HMDB IDs for the 69 known metabolites (16 for replication stage) associated with psoriasis risk in the initial stage and conducted metabolic pathway enrichment analysis on the 52 (11 for replication stage) identifiable compounds. Two stages’ enrichment results highlighted the primary metabolic pathways as Glutathione metabolism, Biosynthesis of unsaturated fatty acids Alpha Linolenic Acid and Linoleic Acid Metabolism (initial stage) and Glycerophospholipid metabolism, Arachidonic acid metabolism (replication stage). Due to the limited amount of metabolites, the SMPDB database was unable to perform enrichment analysis in the replication stage. Specific enrichment results are presented in **Supplementary Table 3** and **Figures 4B, C**.

## 4 Discussion

To our knowledge, there has been no study reported that investigates the causal relationship between serum metabolites and psoriasis using MR analysis. Our study pioneers the systematic exploration of the association between serum metabolites and the risk of psoriasis through MR analysis, complemented by pathway enrichment analysis. We strengthened our preliminary findings with validation analyses and meta-analyses, identifying 17 metabolites (16 known and 1 unknown) causally linked to the risk of developing psoriasis. The known metabolites are predominantly involved in lipid metabolism pathways. Pathway analysis highlighted the importance of glutathione metabolism, alpha-linolenic and linoleic acid metabolism, biosynthesis of unsaturated fatty acids etc. We reviewed the literature on 16 known metabolites and summarized the reported roles of 9 searchable metabolites in different diseases in **Table 1**.

**Table 1:** The known interaction effects of metabolites in different diseases from previous studies.

Metabolite	Biological function	Ref.
Eicosapentaenoate (EPA, 20:5n3)	1.Anti-inflammatory benefits for various diseases. 2.Maintains gut health and immune function. 3.Boosts muscle function and athletic recovery. 4.Protects the heart and prevents blood clots. 5.Reduces lipid oxidation and enhances the effects of statins. 6.Alleviates psoriasis symptoms and lowers lipid toxicity. 7.Reduces inflammation by decreasing IL-17A T cells. 8.Enhances skin lipid balance and promotes skin health.	(21–28)
Docosapentaenoate (n3 DPA, 22:5n-3)	1. Promotes endothelial migration. 2. Regulates gut flora for colitis relief. 3. n-3 DPA reduces macrophage inflammation. 4. Low n-3 DPA correlates with increased CRP and triglycerides. 5. Higher DPA reduces RSV risk in infants.	(29–33)
linoleoyl-arachidonoyl-glycerol (18:2/20:4)	Linoeloyl-arachidonoyl-glycerol (18:2/20:4) correlates positively with serum α-tocopherol concentrations.	(34)
Arachidonate (20:4n6)	1.Regulates blood pressure, diabetes. 2. Crucial for infant growth, neural, immune maturation. 3.Marker for COPD, nephrotic syndrome, prostate cancer. 4.Affects hBMSC immunomodulation.	(35–39)
1-palmitoyl-2-palmitoleoyl-gpc (16:0/16:1)	1. Correlates with increased depression risk. 2. Potential biomarker for diagnosis of colorectal cancer. 3.Aids in surgical boundary definition of oral cancer	(40–42)
3-methylglutaconate	1.Linked to aciduria and congenital metabolic defects in mitochondrial energy metabolism 2.Induces hepatic lipid peroxidation, disrupts redox balance by altering enzymatic/non-enzymatic antioxidants. 3.Reduces non-enzymatic antioxidant defense in brain cortical cells, triggers lipid oxidative damage.	(43–46)
Catechol sulfate	1.Biomarker for ultra-processed food intake. 2.Assesses renal function and dialysis efficacy in CKD.	(47, 48)
Gamma-glutamylvaline	1.Reducing colitis inflammation 2.Rrevents TNF-α-induced inflammatory cytokines in adipocytes, protecting against inflammation-induced insulin resistance 3. Inhibiting the gamma-glutamyl cycle, exhibiting anticancer activity. 4.Suppresses LPS-induced pro-inflammatory cytokines in sepsis. 5.Lowers TNF-α-induced vascular inflammation by activating endothelial CaSR and reducing adhesion molecules and cytokines.	(22, 49–53)
Carnitine C14	1.Induce inflammation via cytokine expression and JNK/ERK phosphorylation. 2.Limits autophagosome-lysosome fusion, reduces autophagy. 3.Correlates with increased risk of diabetic cardiovascular disease. 4.Potential biomarker for conditions like neonatal hypoxic-ischemic encephalopathy, liver injury, knee cartilage volume loss.	(54–60)

Numerous observational studies have identified a pervasive dysregulation of lipid metabolism in patients with psoriasis, and our research has also discerned a causal link between 13 lipid metabolites and the onset of psoriasis. Lipid metabolites such as Docosapentaenoate n3 (DPA 22:5n3), commonly referred to as n-3 DPA, and Eicosapentaenoate (EPA; 20:5n3), known as EPA, are omega-3 polyunsaturated fatty acids. n-3 DPA is an intermediate between EPA and DHA. On the one hand, lower n-3 DPA concentrations have been found to correlate with higher levels of the inflammatory marker CRP (32), and n-3 DPA can reduce the expression of pro-inflammatory factors IL-6, IL-1β in RAW264.7 macrophages stimulated by LPS (33). Therefore, n-3 DPA has a certain anti-inflammatory effect, which may play a role by reducing the inflammatory response of psoriasis lesions. On the other hand, it has been reported that the disturbance of intestinal flora can destroy the intestinal mucosal barrier, increase intestinal mucosal permeability, and reduce the metabolism of probiotics and short chain fatty acids, thus aggravating the intestinal inflammatory response in patients with psoriasis (61). n-3 DPA and EPA play an important role in maintaining the integrity of intestinal barrier, increasing the diversity of intestinal microbiota, and regulating intestinal immunity (23, 29), both may play an important role in the gut-cutaneous axis by maintaining the balance between the gut microbiota, repairing the intestinal barrier, and reducing inflammation. Additionally, EPA has been shown to reduce the proportion of IL-17A producing T-cells and diminish the production of inflammatory mediators by psoriatic keratinocytes and T-cells, thus exerting its anti-inflammatory effects (25, 26). Moreover, supplementation with EPA can increase the content of EPA and n-3 DPA in the dermal and epidermal phospholipids, balance the production of epidermal lipid mediators, promote normal differentiation of psoriatic epidermis, and restore homeostasis at psoriatic skin lesions (25). The elevated levels of DPA and EPA found in our study correlate with a reduced risk of psoriasis, aligning with previous research. Supplementation of DPA and EPA may mitigate the risk of psoriasis by regulating intestinal immunity, modulating the balance of the gut microbiome, inhibiting inflammatory responses, and promoting epidermal differentiation.

Gamma-glutamylvaline (γ-EV) is a dietary peptide ubiquitously found in various foods. As demonstrated in **Table 1**, γ-EV has been identified to play a role in multiple inflammatory animal and cell models, such as colitis in murine intestinal epithelial cells, adipocytes, and human aortic endothelial cells (50, 52, 62). Additionally, research by Chee et al. has shown that oral administration of γ-EV can reduce the expression levels of pro-inflammatory cytokines TNF-α, IL-6, and IL-1β in the serum and small intestine of sepsis-induced mice by LPS, exhibiting anti-septic systemic inflammatory activity (22) Zhang and colleagues have also discovered that supplementation with γ-EV can alleviate intestinal inflammation in a porcine model of colitis (51). Although there is no explicit research evidence directly linking γ-EV with psoriasis or its impact, psoriasis is globally recognized as a chronic inflammatory skin disease, and the interplay of inflammation and response is a crucial pathogenic mechanism. Our study results have found Gamma-glutamylvaline (γ-EV) to be a protective factor for psoriasis (OR:0.71, 95%CI:0.59-0.85, *P*<0.001). γ-EV may exert a therapeutic effect by reducing the level of inflammation in epidermal cells. Therefore, the supplementation of γ-glutamylvaline could potentially be beneficial for psoriasis management.

Our study also identified exogenous metabolite catechol sulfate levels and the amino acid metabolite 3-methylglutaconate levels as risk factors for the onset of psoriasis. Catechol sulfate is recognized as one of the biomarkers for the intake of ultra-processed foods, with its metabolite levels significantly correlating with the consumption of such foods (48) The increased consumption of ultra-processed foods has been reported to elevate the risk of various diseases, including diabetes, depression, and cancer (63–65). Therefore, individuals with psoriasis should limit their intake of ultra-processed foods to prevent the potential risk increase associated with high levels of catechol sulfate metabolites. Dysregulation of 3-methylglutaconic acid metabolism has been noted in conditions such as 3-methylglutaconic aciduria and various congenital metabolic defects where mitochondrial energy metabolism is impaired (44, 45). Elevated levels of 3-methylglutaconic acid detected in various inherited metabolic diseases could lead to dysfunctions in the nervous system, heart, liver, and other organs (66, 67). Leipnitz and colleagues have further found through *in vitro* experiments that the accumulation of 3-methylglutaconic aciduria metabolites can increase oxidative stress in cerebral cortical epithelial cells, potentially contributing to mechanisms inducing brain damage (46). At present, there are no studies on the correlation between these two metabolites and psoriasis, and our results confirm that increased levels of catechol sulfate and 3-methylglutaconate increase the risk of psoriasis, but further mechanism studies are still to be conducted.

Additionally, we found two metabolites that are risk factors in other diseases, yet are protective factors in our results. As demonstrated in **Table 1**, research by van et al. has indicated a positive correlation between the levels of 1-palmitoyl-2-palmitoleoyl-gpc (16:0/16:1) and the risk of depression (42). Although psoriasis patients often have mental illnesses such as depression, our results suggest a negative correlation between 1-palmitoyl-2-palmitoleoyl-gpc (16:0/16:1) levels and the incidence of psoriasis. Carnitine C14 may activate pro-inflammatory pathways to induce inflammatory responses and is positively associated with the risk of diabetic cardiovascular disease (56, 58), suggesting that Carnitine C14 may be a potential risk factor, which is inconsistent with our results. This could potentially be due to a bias from the single SNP obtained under the current screening criteria, therefore, it is necessary to relax the screening criteria to include more SNPS to increase the interpretation of phenotypes. Of course, in order to better understand the role of metabolites in psoriasis, further clinical research or experimental verification is more necessary.

It is noteworthy that the metabolites identified in our study results are primarily enriched in glutathione metabolism, as well as alpha-linolenic and linoleic acid metabolism pathways. Glutathione is a vital metabolic regulator with intracellular antioxidative and anti-inflammatory functions, playing a role in maintaining normal immunity. Oxidative stress markers are established to be elevated in psoriasis and correlate with the disease’s course and severity (68), which may be associated with the abnormal levels of the antioxidant glutathione in the skin lesions and peripheral blood of patients with psoriasis, as well as dysregulation of related transferase activities (69). Furthermore, basic experimental evidence confirms that levels of antioxidant markers such as glutathione (GSH) and superoxide dismutase (SOD) are decreased in psoriasis mouse and cell models, which are reversed upon treatment (70, 71). Impairment of glutathione metabolism in psoriasis may relate to an imbalance in the oxidative-antioxidative system, inflammatory responses, and immune system dysregulation. Research reveals a strong positive correlation between glutathione and inflammatory cytokines, angiogenic initiators in psoriasis (72), and it plays a significant role in various psoriasis-associated cells. Liu et al. found increased numbers of MDSCs and M-MDSCs in the peripheral blood and skin lesions of psoriasis patients, and acitretin promotes the differentiation of MDSCs by increasing the expression of glutathione synthetase (GSS) and accumulation of glutathione (GSH), thus reducing ROS levels (73). Campione et al. reported increased activity of glutathione-S-transferase (GST) in the lesional areas of psoriasis, exerting anti-inflammatory effects in the hyperproliferative keratinocytes characteristic of psoriasis (69). Additionally, GSH can regulate the expression levels of IκBζ in macrophages (74). The oxidative stress levels in dendritic cells and neutrophils are increased in the IMQ-induced psoriasis model (72). In conclusion, glutathione metabolism plays an important role in the pathological processes of psoriasis such as oxidative stress and inflammation by affecting a variety of cells, such as keratinocytes, macrophages, MDSC, and dendritic cells.

Alpha-linolenic acid (ALA) and linoleic acid (LA), as essential polyunsaturated fatty acids, play a pivotal role in human metabolism. Vahlquist et al. observed significant reductions in the levels of linoleic acid (18:2 omega-6) and α-linolenic acid (18:3 omega-3) in the plasma lipid esters of patients with psoriasis compared to a healthy control group. Notably, these reductions were even more pronounced in patients with severe psoriasis, suggesting a possible correlation between fatty acid levels and disease severity (75). Methotrexate, a cornerstone medication in psoriasis treatment, has been proven to modulate linoleic acid metabolism within CD4^+^ central memory T cells and CD8^+^ effector memory T cells (76). Experimental studies have elucidated that ALA and LA, under the action of lipoxygenase and cyclooxygenase, give rise to eicosanoids such as prostaglandins (PGs), thromboxanes, and leukotrienes (LTs), which play crucial roles in inflammation and hemodynamic regulation, mediating inflammatory and allergic diseases like psoriasis and atopic dermatitis (76–78). In metabolic pathways associated with psoriasis, linoleic acid exhibits a negative correlation with AMPK and the PI3-Akt signaling pathway. Specifically, ALA has been shown to mediate the Ras signaling pathway, participating in cell adhesion and the transendothelial migration of leukocytes (79). Moreover, ALA interacts directly with the NF-κB pathway and, as a ligand for PPARs, expresses bioactivities with anti-inflammatory capacities (80, 81). The perspective of Simopoulos on dietary intake ratios reveals the inflammatory impact of a high n-6 to n-3 fatty acid ratio (20:1) prevalent in Western diets, which may enhance the production of pro-inflammatory mediators. In contrast, a more balanced intake ratio (approximately 1:1) is considered to have protective effects against inflammation (82). Synthesizing these insights regarding the metabolic pathways of ALA and LA unveils their anti-inflammatory attributes and accentuates the nuanced effects of varying dietary intake ratios. This body of evidence provides compelling justification for further investigation into the therapeutic potential of modulating these fatty acid levels in the management of inflammatory diseases.

In addition, unsaturated fatty acid related metabolites EPA and n-3 DPA have been discussed above. Many elements in the glycerol phospholipid metabolism pathway, such as PA, PC, and PI, have also been confirmed to undergo significant changes in the plasma of psoriasis patients (4). Drugs such (R)-salbutamol and Chinese Herbal formula can alleviate psoriasis by improving glycophoric metabolism (83, 84). Arachidonic acid (AA) is a polyunsaturated fatty acid that serves as a precursor to various bioactive lipid mediators, including prostaglandins, leukotrienes, and thromboxanes. These eicosanoids are potent regulators of inflammation and immune responses. In psoriasis, the metabolism of arachidonic acid is often dysregulated. Clinical studies have found that compared with female psoriasis patients, psoriatic skin lesions are more severe in male patients and plasma AA concentration is significantly lower than that in female patients. AA is positively correlated with DLQI score, which may be related to estrogen metabolism (85). Ye et al. reported that CD4^+^ T cells in patients with psoriatic arthritis (PsA) increased the expression of the poro-forming calcium channel component ORAI3, thereby increasing the activity of calcium selective channels regulated by arachidonic acid, making T cells sensitive to arachidonic acid and contributing to the chronic inflammatory response of PsA (86). In addition, inflammatory mediators such as PGE2 and LTB4 produced by Arachidonic acid and its derivatives catalyzed by cycoperoxidase can recruit and activate T cells to participate in inflammation (26, 87).Therefore, arachidonic acid and its derivatives may play a key role in the pathogenesis of psoriasis by influencing the function and sensitivity of T cells and activating the inflammatory response.

Our study has several limitations: Firstly, we used strict screening conditions (*P*< 5×10^-8^, r^2<^ 0.001 within a 10000 kb distance) and the SNPS of many metabolites could not be extracted. After we broadened the screening criteria (*P<* 5×10^-7^, r^2^< 0.01 within a 5000kb distance), some metabolites still obtained fewer SNPs under the currently set screening conditions, which may lead to a bias in the results. Secondly, many metabolites filtered during the initial stage failed to achieve significance post-rigorous multiple testing adjustments. Thirdly, the population data comes from European ancestry, and there may be differences when the findings generalize to other ethnicities. Therefore, more people with different genetic backgrounds should be analyzed to improve the generality of the results. Lastly, the employment of genetic variants linked to metabolites as instrumental variables reflects prolonged exposure scenarios; conversely, transient dietary supplementation may elicit disparate effects on the delineated results.

In conclusion, our study found that 24 metabolites (7 metabolite ratios, 16 known metabolites, and 1 unknown metabolite) are associated with psoriasis risk, and most of these metabolites belong to lipid metabolism. These metabolites may be biomarkers to predict the onset and development of psoriasis. This study also provides recommendations for dietary adjustment and nutritional intervention in clinical psoriasis patients, such as increasing the intake of γ-EV or Omega-3 fatty acids such as DPA, EPA, and reducing the intake of ultra-processed foods to reduce the production of catechol sulfate have a positive effect on the control of psoriasis.

## Data Availability

The original contributions presented in the study are included in the article/**Supplementary Materials**, further inquiries can be directed to the corresponding authors.
