# Corrigendum: The causal relationship between serum metabolites and the risk of psoriasis: a Mendelian randomization and meta-analysis study

**DOI:** 10.3389/fimmu.2024.1544371

**Published:** 2025-01-06

**Authors:** Yujie Yang, Xuwei Zheng, Haiying Lv, Bin Tang, Yiyuan Zhong, Qianqian Luo, Yang Bi, Kexin Yang, Haixin Zhong, Haiming Chen, Chuanjian Lu

**Affiliations:** ^1^ The Second Clinical College of Guangzhou University of Chinese Medicine, Guangzhou, China; ^2^ State Key Laboratory of Dampness Syndrome of Chinese Medicine, The Second Affiliated Hospital of Guangzhou University of Chinese Medicine (Guangdong Provincial Hospital of Chinese Medicine), Guangzhou, China; ^3^ Guangdong Provincial Key Laboratory of Clinical Research on Traditional Chinese Medicine Syndrome, Guangzhou, China; ^4^ Guangdong Provincial Clinical Medicine Research Center for Chinese Medicine Dermatology, Guangzhou, China; ^5^ Guangdong-Hong Kong-Macau Joint Lab on Chinese Medicine and Immune Disease Research, Guangzhou University of Chinese Medicine, Guangzhou, China

**Keywords:** psoriasis, Mendelian randomization, metabolites, causal effect, implication

In the published article, there was an error in **Figure 4** as published. The duplicate **Figure 4B** was mistakenly uploaded to the position of **Figure 4C**. The corrected **Figure 4** and its caption appear below.

**Figure 4:**
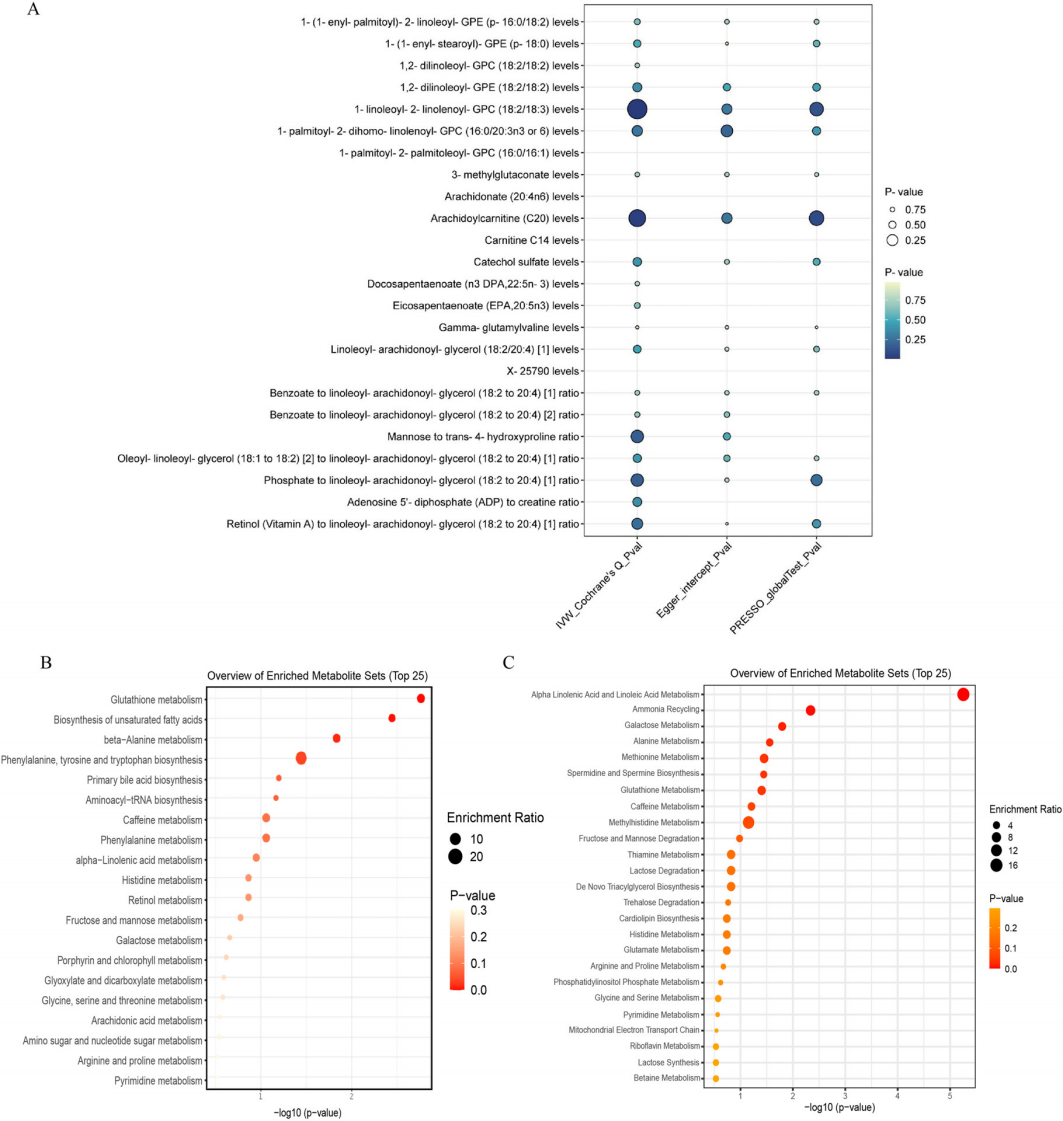
**(A)** displays the heterogeneity and pleiotropy analysis results of 24 metabolites that have been successfully validated. **(B)** displays the enrichment pathways of metabolites in KEGG and **(C)** displays the enrichment pathways of metabolites in SMPDE database.

The authors apologize for this error and state that this does not change the scientific conclusions of the article in any way. The original article has been updated.

